# Supplementary material for: Comparative Stability Study of Polysorbate 20 and Polysorbate 80 Related to Oxidative Degradation
Source: Pharmaceutics. 2023 Sep 16;15(9):2332. doi: 10.3390/pharmaceutics15092332 (PMC10537708; doi:10.3390/pharmaceutics15092332)
Supplement: Supplementary file 1 [file pharmaceutics-15-02332-s001.zip › pharmaceutics-2542210-supplementary.pdf]

Supplementary Information:

Comparative Stability Study of Polysorbate 20 and Polysorbate 80 Related to Oxidative Degradation

Benedykt Kozuch[1], Johanna Weber[2], Julia Buske[1], Karsten Mäder[2], Patrick Garidel[1*], Tim Diederichs[1*]

[1]Boehringer Ingelheim Pharma GmbH & Co. KG, Innovation Unit, TIP, Birkendorfer Straße 65, 88397 Biberach an der Riss, Germany

[2]Martin-Luther-University Halle-Wittenberg, Institute of Pharmacy, Faculty of Biosciences, Wolfgang-Langenbeck-Strasse 4, 06120 Halle (Saale), Germany

*Corresponding author:

Tim Diederichs: tim.diederichs@boehringer-ingelheim.com

Patrick Garidel: patrick.garidel@boehringer-ingelheim.com

Boehringer Ingelheim Pharma GmbH & Co. KG, Innovation Unit, TIP, 88397 Biberach an der Riss, Germany

Calculation molar ratios oxygen vs. polysorbate

With the given headspace volume (5 mL), the density of oxygen gas under standard conditions (0.001429 $g \cdot cm^{-3}$), the content of oxygen in the atmosphere (0.20942 (v/v)), and the atomic weight of oxygen (32 Da), the molar amount of oxygen in the headspace of a single vial can be estimated to be approximately 0.047 mmol. The dissolved oxygen in the 5 mL solution can be calculated using the solubility of oxygen under standard conditions ($2.56 \cdot 10^{-4}$ $mol \cdot dm^{-3}$), giving approximately 0.00128 mmol. Therefore, approximately 0.048 mol oxygen is available per vial. With the molecular weights of 1228 $g \cdot mol^{-1}$ for polysorbate 20 (PS20) and 1310 $g \cdot mol^{-1}$ for polysorbate 80 (PS80), 0.041 mmol and 0.038 mmol of PS material were calculated for 10 $mg \cdot mL^{-1}$ PS20 and PS80 formulations. For 0.2 $mg \cdot mL^{-1}$ PS20 and PS80 0.814 µmol and 0.763 µmol, respectively.

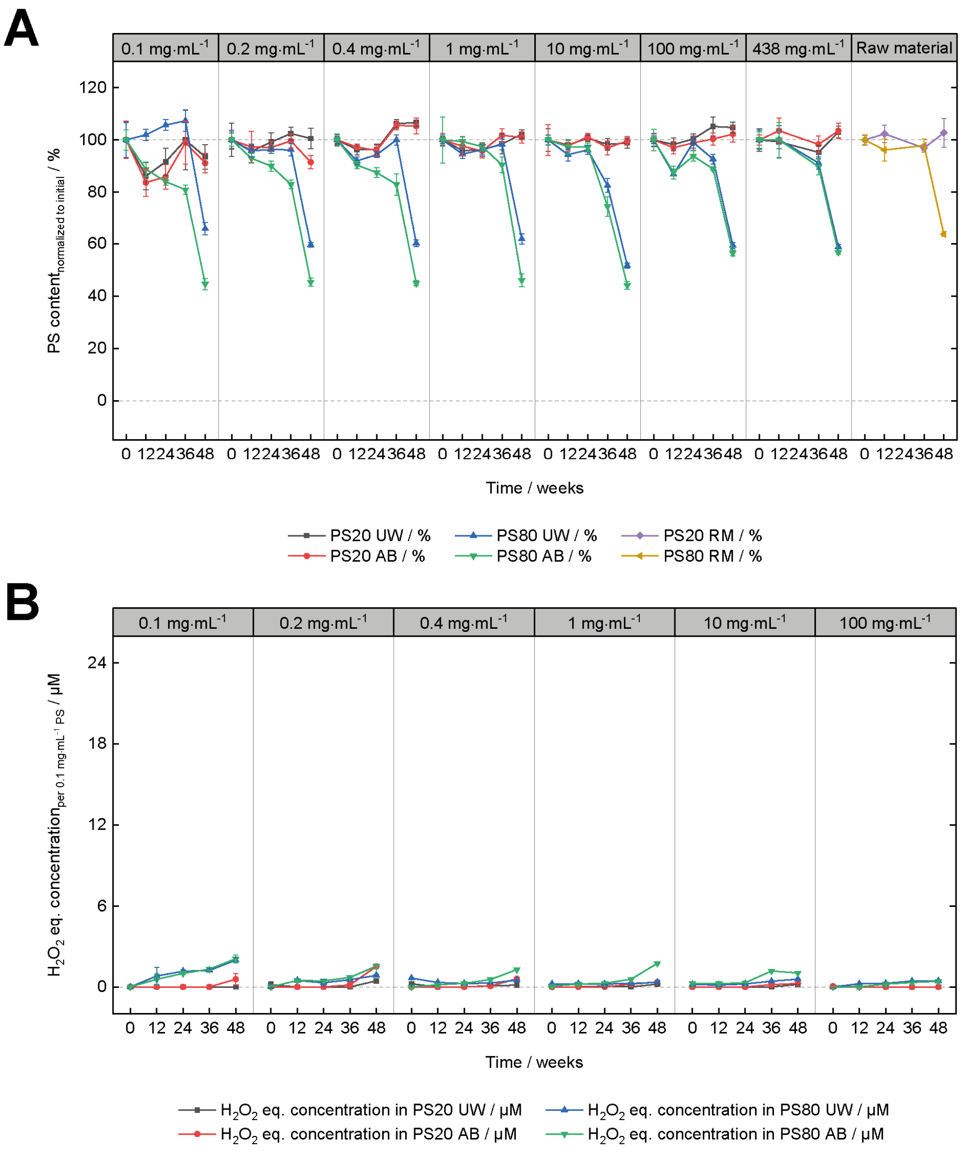

Figure S1. FMA (fluorescence micelle assay) and FOX (ferrous oxidation with xylenol orange) measurements of polysorbate formulations stored at 5 °C. (A) PS20 and PS80 contents were determined for different initial PS concentrations in water (UW) and 25 mM acetate buffer (AB) pH 5.5 stored for 48 weeks at 5 °C *via* FMA. The data were normalized to the initially measured PS concentration of each formulation. Two biological replicates of each sample (n = 2, except for the raw materials) were measured with four technical replicates each (n = 4). The reference lines mark PS concentrations of 0 and 100 % of the initial values. **(B)** H_2O_2 equivalent concentrations for PS20 and PS80 concentrations of 0.1 – 100 mg·mL^{-1} PS formulated in UW and AB pH 5.5 for the storage of 48 weeks at 5 °C measured *via* the FOX assay. The data are presented as H_2O_2 equivalent concentration per 0.1 mg·mL^{-1} PS in the formulations. Three technical replicates of each sample were measured (n = 3). The reference line marks a H_2O_2 equivalent concentration of 0 µM.

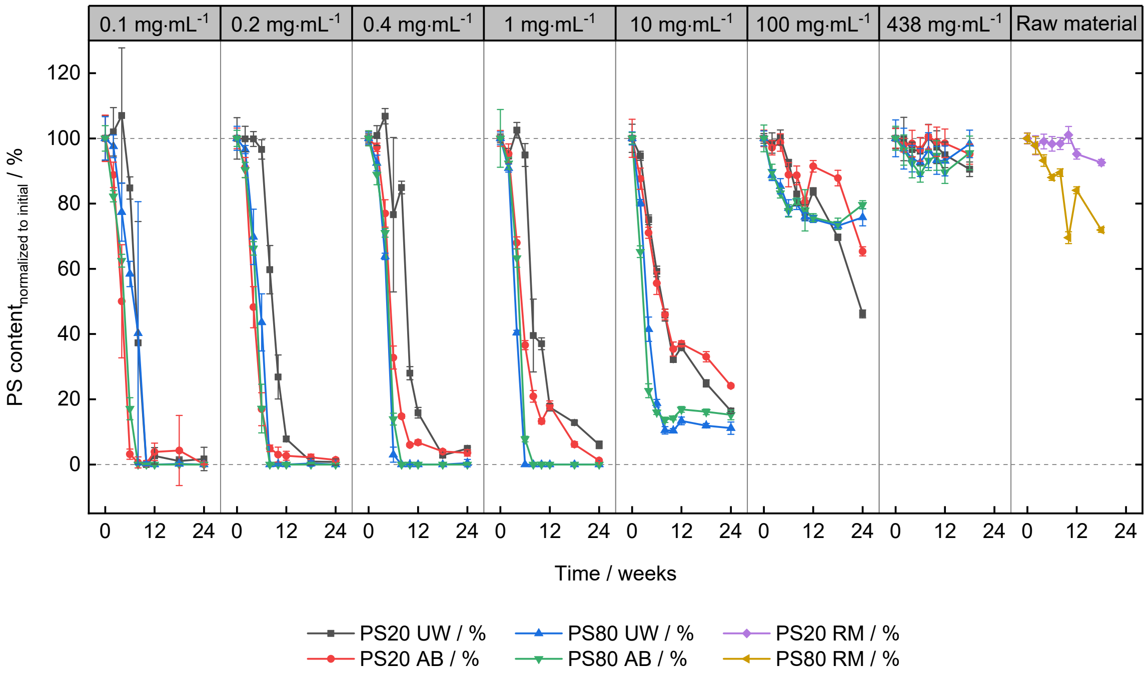

Figure S2. Zoom in on FMA (fluorescence micelle assay) measurements of polysorbate formulations stored at 40 °C for better visualization of the lag phase in PS20 UW formulations. PS20 and PS80 contents were determined for different initial PS concentrations in water (UW) and 25 mM acetate buffer (AB) pH 5.5 stored for 48 weeks at 40 °C/75 % rh *via* FMA. The data were normalized to the initially measured PS concentration of each formulation. Two biological replicates of each sample ($n = 2$, except for the raw materials) were measured with four technical replicates each ($n = 4$). The reference lines mark PS concentrations of 0 and 100 % of the initial values. Only data up to 24 weeks are illustrated.

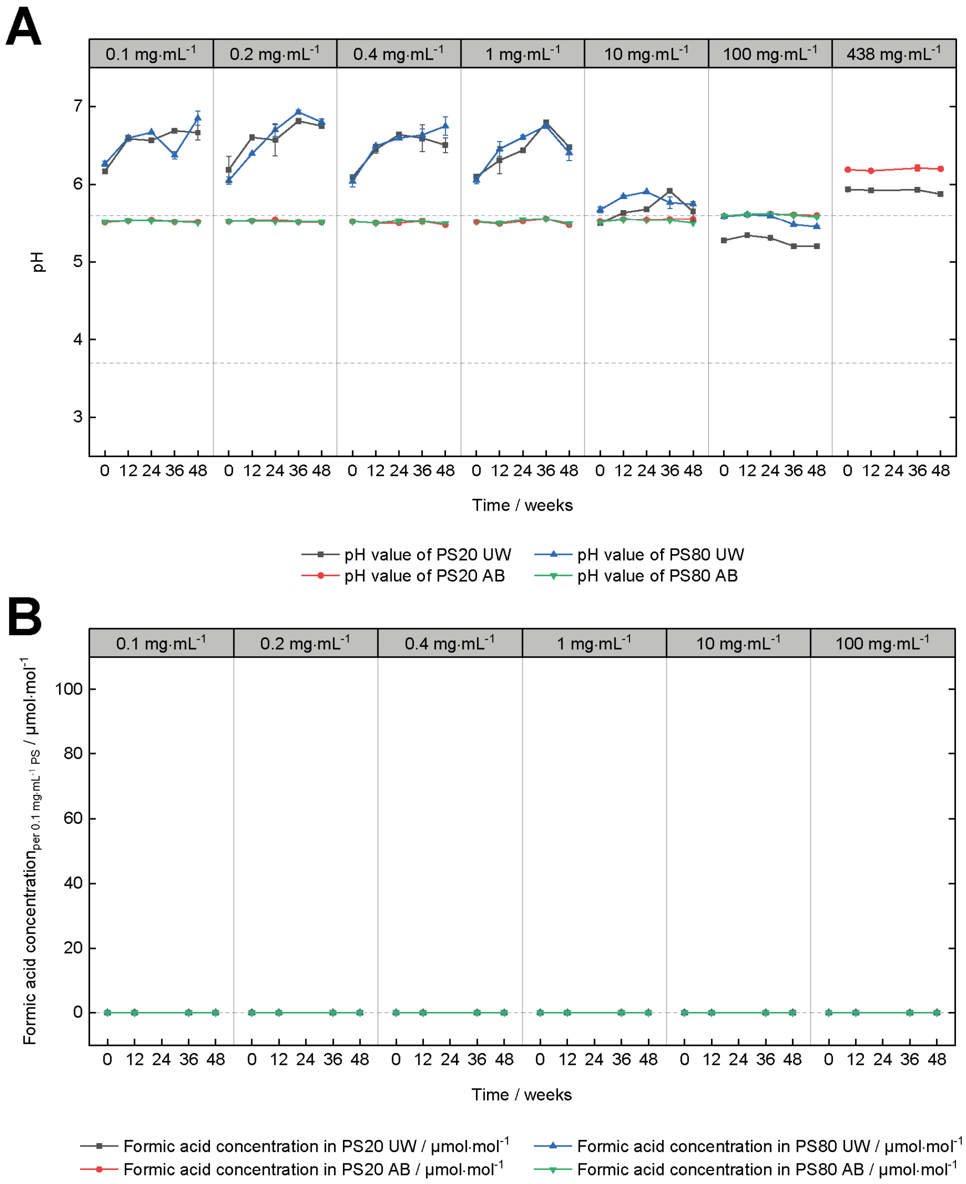

Figure S3. pH and GC (gas chromatography) measurements of polysorbate formulations stored at 5 °C. (A) pH values of all PS formulations formulated in water (UW) and 25 mM acetate buffer (AB) pH 5.5 used in the stability study after 48 weeks of storage at 5 °C. Two biological replicates of each sample were measured (*n* = 2). 438 mg·mL^{-1} PS80 formulations and PS raw material samples were not measured. Reference lines mark the buffer range of the used 25 mM acetate buffer (pH 3.7 – 5.6). **(B)** Formic acid concentrations for PS formulations in UW and AB pH 5.5 for PS concentrations of 0.1 – 100 mg·mL^{-1} used in the stability study after 48 weeks of storage at 5 °C measured *via* GC. The data are presented as formic acid concentration per 0.1 mg·mL^{-1} PS in the formulations. One replicate of each sample was measured (*n* = 1). 438 mg·mL^{-1} PS formulations and PS raw material samples were not measured. The reference line marks a formic acid concentration of 0 µmol·mol^{-1}.

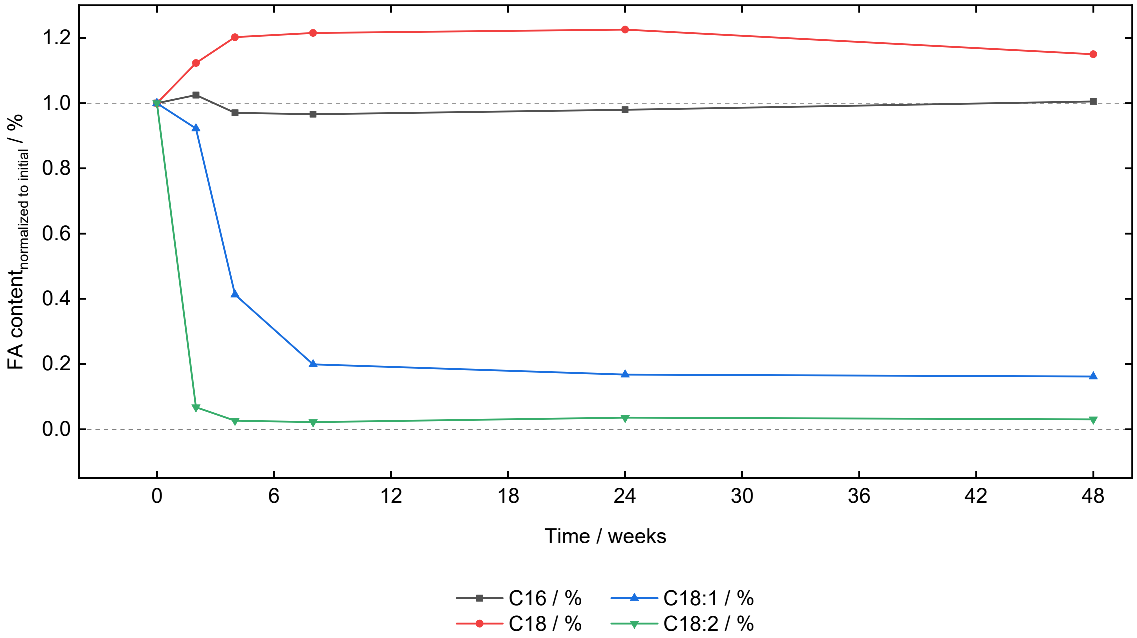

Figure S4. Preferential oxidation of PS80 esterified to different fatty acids. The peak patterns of 0.4 g·mL^{-1} PS80 AB at 40 °C, were used to extract the intensities the different polyoxyethylene variants for palmitic acid (C16), stearic acid (C18), and oleic acid (C18:1). The intensities are normalized to the corresponding initial values (t = 0) and plotted versus time. For C18 only approximately 2% was initially present in the raw material of PS80, resulting in low signal intensities with low signal-to-background values. The observed increase for C18 is must likely based on the inaccurate determination of the initial timepoints.

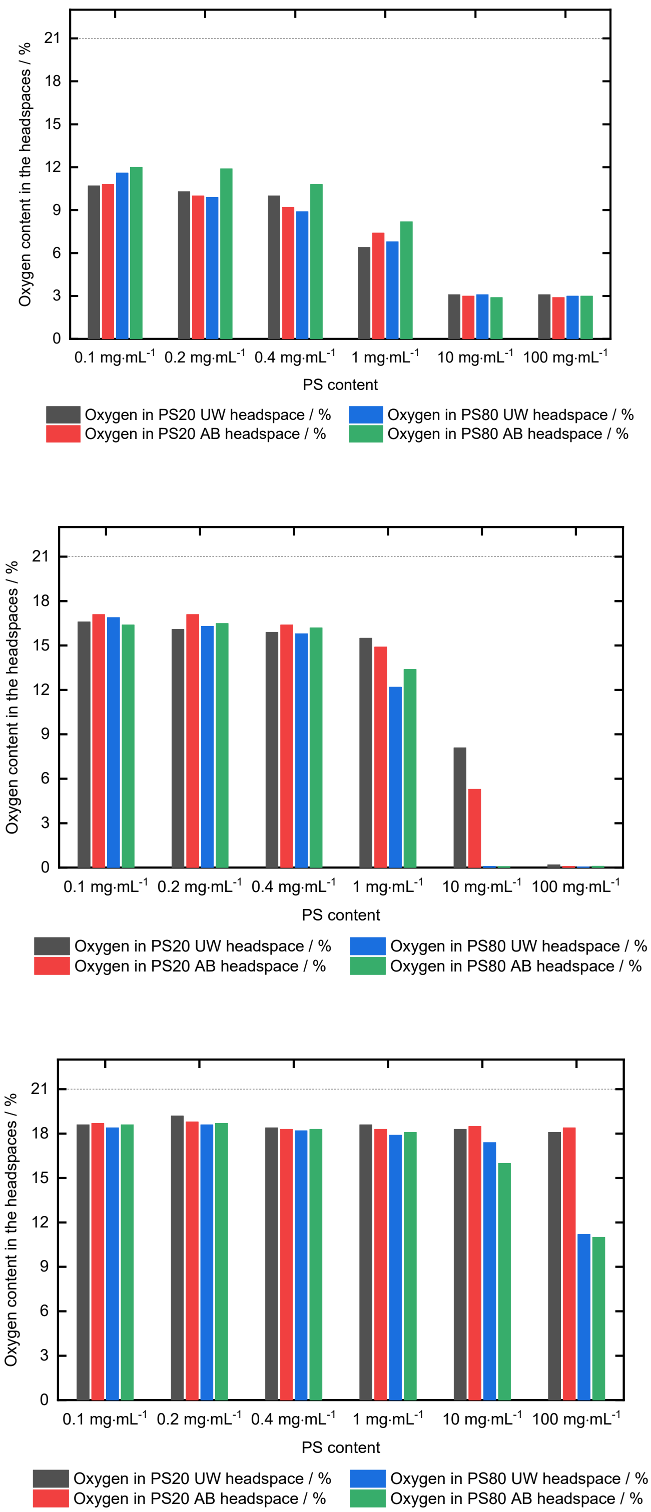

Figure S5. Oxygen content in vials after storage for 48 weeks at 40, 25, and 5 °C. The oxygen content in the vials after 48 weeks at the different storage temperatures were measured in vials after 48 weeks of storage with the Microx 4 trace fiber optic oxygen meter. The upper, middle, and bottom panels show the oxygen values for 40, 25, and 5 °C, respectively.

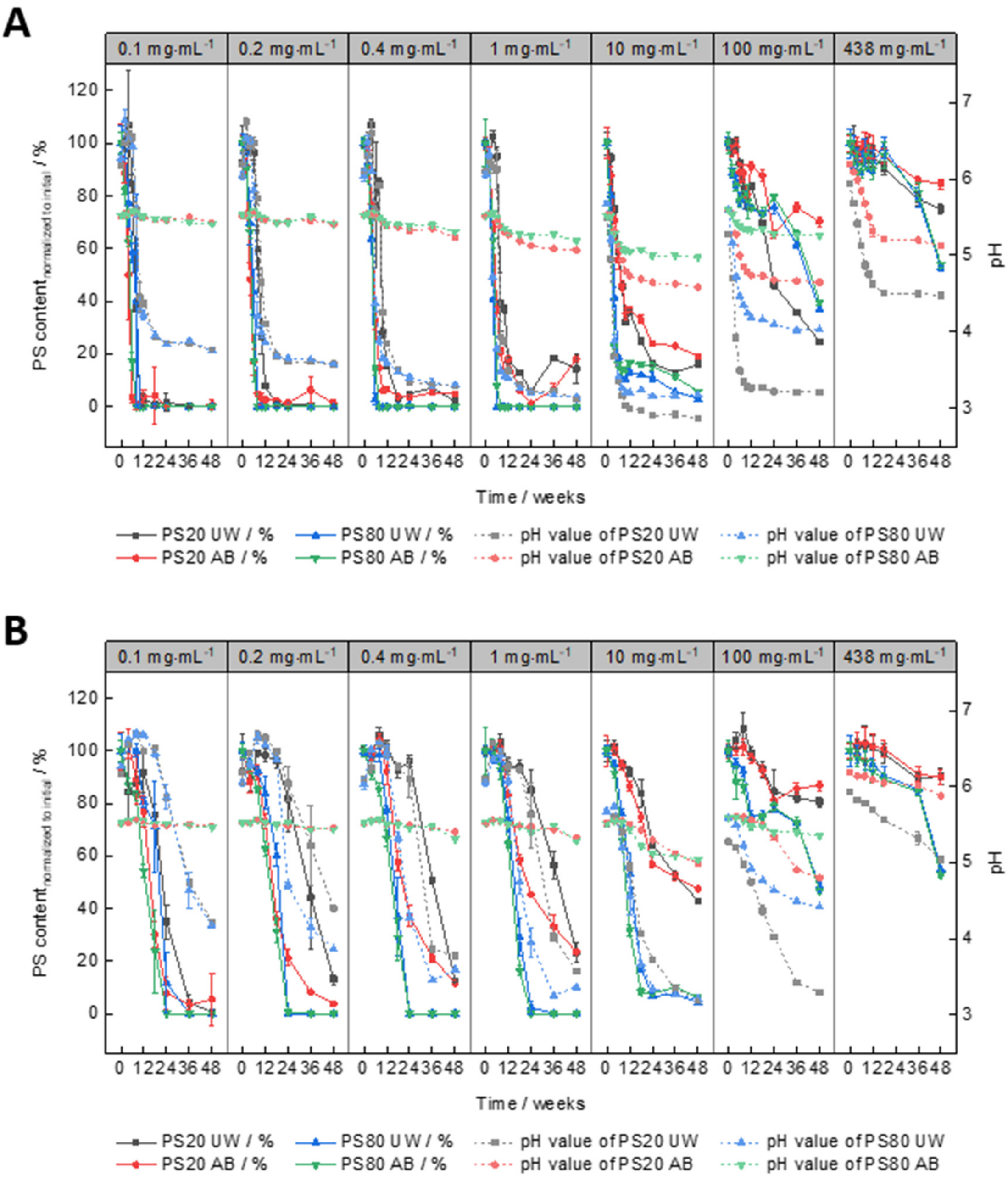

Figure S6. Overlay of PS content (FMA) and pH for the storage of 40 and 25 °C. (A/B) PS20 and PS80 contents and pH values were determined for different initial PS concentrations in water (UW) and 25 mM acetate buffer (AB) pH 5.5 stored for 48 weeks at 40 °C/75 % rh (A) and 25 °C/60 % rh (B). FMA data were normalized to the initially measured PS concentration of each formulation. Two biological replicates of each sample (n = 2, except for the raw materials) were measured with four technical replicates each (n = 4). The reference lines mark PS concentrations of 0 and 100 % of the initial values. pH values of 438 mg·mL^{-1} PS80 formulations and PS raw material samples were not measured. Reference lines mark the buffer range of the used 25 mM acetate buffer (pH 3.7 – 5.6).
